# Superficial Tunica Albuginea Rupture as Initial Starting Point of Peyronie's Disease: A Topic for Interdisciplinary Consideration

**DOI:** 10.1155/2015/751372

**Published:** 2015-01-15

**Authors:** Ralf Herwig, Manfred Bayerl

**Affiliations:** ^1^Department of Urology, Vienna International Medical Clinic, Antonigaße 97/6, 1170 Vienna, Austria; ^2^International Study Group on Self-Organisation, Morizgasse 4, 1060 Vienna, Austria

## Abstract

Peyronie's disease is a connective tissue disorder in the soft tissue of the penis. The underlying cause of Peyronie's disease is not well understood but is thought to be caused by trauma or injury to the penis during sexual intercourse. The purpose of the interdisciplinary cooperation between urological surgery and physics is the development of a physical simulation tool in order to give prognosis of possible tunica albuginea fibre rupture at a certain degree of deviation of the penis. For our group the first challenge was to translate the human organ of the penis into a physical model. Starting and marginal parameters had to be defined, whereby some of them had to be based on assumption, as physical data of the human living tissue have rarely been measured up to now. The algorithm and its dependencies had to be developed. This paper is a first step of a three-dimensional mathematical-physical simulation with the assumption of a 100% filled rigid penis. The calculation gives proof of the hypothesis that the fibre-load-angle of the penis is less than 12 degrees. Thus physical simulation is able to provide the surgeon with a simple instrument to calculate and forecast the risk of the individual patient.

## 1. Introduction

Peyronie's disease, also known as induratio penis plastica [1] or chronic inflammation of the tunica albuginea (CITA), is a connective tissue disorder involving the growth of fibrous plaques [1] in the soft tissue of the penis affecting about 5% of men in mean [1], whereas the data vary from 0.6 to 23% [2–6] of all men.

Specifically, scar tissue forms in the tunica albuginea, the thick sheath of tissue surrounding the corpora cavernosa causing pain, abnormal curvature, erectile dysfunction, indentation, loss of girth, and shortening.

The underlying cause of Peyronie's disease is not well understood but is thought to be caused by trauma or injury to the penis usually through sexual activity although many patients often are unaware of any traumatic event or injury [7].

After traumatic event, Peyronie's disease (PD) is a progressive fibrotic disorder of the penis that is characterized by formation of collagen plaques on the tunica albuginea of the penis that may result in penile deformity and pain (typically early in the disease course) and often occurs in conjunction with erectile dysfunction [1, 7, 8].

A possible underlying cause of trauma has not been investigated so far.

We started an interdisciplinary cooperation between medicine and mathematical physics to create a mathematical model of the penis, which provides the possibility to perform measurements to investigate the mechanism of initial injury of the superficial tunica.

Mathematical physics is called up to provide a time-dependent model for the surgeon, which enables him consider the individual case in a physical model and thus be able to obtain an exact mathematical prognosis according to the individual geometric conditions of the singular patient.

A medical-physical cooperation in connection with the increasingly problematic situation of penis rupture firstly means for the physicist the problem of finding apt starting and marginal conditions for the conversion of the human organ into a physical model, especially so, as physical material data of the biological tissues are more or less unknown in literature or only in different questions and exact physical measurements of living tissue have not been possible up to now. Thus a certain extent of uncertainty has to be taken into consideration. A physical system at best only approximately fulfills the mathematical conditions on which precise proof is built and a physicist should be constantly aware of the approximate nature of his calculations. Nevertheless, the more precise and the nearer to reality of nature the starting and marginal conditions can be defined, the better the model and the significance of the results will be.

Physically spoken the human organ of the penis is to be defined with its physical (mostly elastic-mechanical) properties, its structural build-up and the properties of the differential geometrical body.

The geometry of the penis, simplified as differential geometrical body, the velocity of stress, and the sort of stress that means tension, pressure, buckling, bending, or thrust, are of decisive importance for a penis rupture [9]. In this work, in the first stage of investigation, we dealt with the buckling stress only.

In case of dynamic stress of the penis inner tensions arise, leading to plastic deformations and, with exceeding the maximum solidity, to notches and ruptures. Apart from the inner tensions there also arise large pressures in the inner layers of the penile tissues. With superimposition of external mechanical stresses and internal tensions the solidity of the fascial tissue is strongly affected.

The fibre-load-angles (longitudinal/vertical to the fibre) and the angles between radial and tangential direction are of considerable influence for the solidity of the penis. These angles have to be determined depending on the elastic-mechanical properties. It is the aim of this work to define the maximum stress loads for the tissue under different forces, different angles, and different velocities of impact.

The underlying work is a first stage of the development of a physical model, as expressive as simply to be handled by surgeons, which is able to deliver prognosis for the individual case on the basis of simply and also in the operating theatre to be measured geometrical conditions (length, circumference, and geometrical angle) of the singular penis. Thus it is necessary to develop the algorithm for these dependencies.

## 2. Method and Approach

In developing a physical model for the use of surgeons to be varied for the individual patient two questions arose for the physicist, the microscopic approach of the buckling stress, analytically, with the view on the singular fibre of penile tissue as well as on *n*-layers, resulting in an analytical algorithm, into which the different geometric conditions of the individual patient can be inserted to gain a critical degree of deviation and the macroscopic approach, where a time-dependent model, in which the penis is subdued to an external force, results in the localisation of the point of the greatest stress of the tissue, where a penis rupture is most likely to happen.

After choosing the coordinate system, the directions of stress (longitudinal, radial, and tangential), the cutting planes (tangential plane, cross section, and radial plane), the elastic-mechanical properties of the penis can be defined.

## 3. Elastic-Mechanical Properties of the Penis

**Figure 1:**
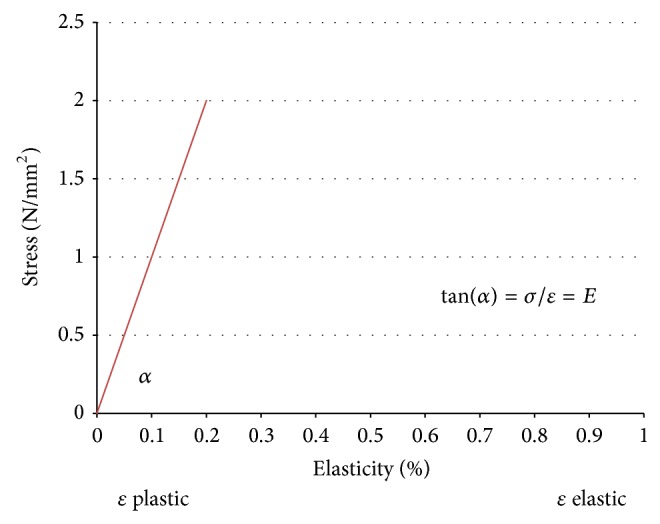
Diagram of stress (*σ*) and elasticity (*ε*).

The elasticity of the fascia tissue of the penis is the capacity to work against a deformation that is effected by external forces. If the penis (the penile tissue) totally regains its original form after release, this is the case of an ideally elastic body. There is a linear relation between stress and elasticity in an ideally elastic penis. This is shown in Figure 1, the diagram of stress and elasticity. The diagram shows a plastic and an elastic range.

Figure 1 shows a diagram of stress and elasticity, which has still to be verified by experiments. According to the literature no tensile or pressure tests are known to be carried out on living tissue. This would still have to be allowed by the ethic commission.

Very simplified the penis can be looked on as orthotropic system with three main axes, in longitudinal (L), radial (R), and tangential (T) direction. According to the theory of orthotropic elasticity, the generalising Hooke's Law is effective. Starting points for the generalisation of Hooke's Law to the three-dimensional state of stress and distortion are positive stresses and distortions in penile tissue, whereby the edges are parallel to the reference system. The tensors of stress and distortion are assumed to be symmetric tensors.

Based upon inhomogeneity and anisotropy the access and the definition of the properties of the penis are strongly depending on the direction of stress and the reference plane, respectively.

## 4. Microscopic Approach

The analytical microscopic approach is based on the physical dependencies and relations and the geometric and elastic-mechanical properties of the penis. In the elastic range of the diagram of stress and elasticity Hooke's Law is effective.

Using the elasticity matrix *S* for the orthotropic body of the penis (direction dependence of the properties along *x*-, *y*-, and *z*-axis) the following matrix shows the dependencies:

(1) $$\begin{array}{c} \left[\begin{array}{c} \varepsilon_{1}\\ \varepsilon_{2}\\ \varepsilon_{3}\\ \gamma_{2,3}\\ \gamma_{1,3}\\ \gamma_{1,2} \end{array}\right]=\left[\begin{array}{cccccc} \frac{1}{E_{1}} & -\frac{\mu_{2,1}}{E_{2}} & -\frac{\mu_{3,1}}{E_{3}} & 0 & 0 & 0\\ -\frac{\mu_{1,2}}{E_{1}} & \frac{1}{E_{2}} & -\frac{\mu_{3,2}}{E_{3}} & 0 & 0 & 0\\ -\frac{\mu_{1,3}}{E_{1}} & -\frac{\mu_{2,3}}{E_{2}} & \frac{1}{E_{3}} & 0 & 0 & 0\\ 0 & 0 & 0 & \frac{1}{G_{2,3}} & 0 & 0\\ 0 & 0 & 0 & 0 & \frac{1}{G_{1,3}} & 0\\ 0 & 0 & 0 & 0 & 0 & \frac{1}{G_{1,2}} \end{array}\right]\cdot \left[\begin{array}{c} \sigma_{1}\\ \sigma_{2}\\ \sigma_{3}\\ \tau_{2,3}\\ \tau_{1,3}\\ \tau_{1,2} \end{array}\right], \end{array}$$

whereby there are *ε*: normal stresses, *σ*: tensor of stress, *τ*: shear stresses, *γ*: tensor of distortion, *E*: modulus of elasticity, *μ*: Poisson ratio, and *G*: modulus of shear.

## 5. Buckling Stress

If buckling stress is applied to a penis with total inner pressure (200 mmHg = 26,6 kPa), a fibre-load-angle *φ* arises, causing bulged sides (dodging). The fibre-load-angle is the geometrical angle between the direction of stress, where the force **F** is effective (pressure, tension), and the direction of the fibre. The force of buckling is effective in the geometric midpoint of the simplified assumption of the cylinder. A strong decrease of solidity arises with the increasing geometrical fibre-load-angle *φ*. The solidity of the penis increases with increasing density and increasing inner pressures.

**Figure 2:**
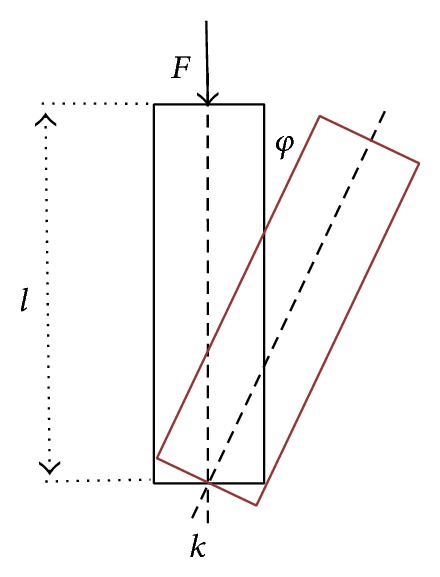
Simplified model of buckling.

A simplified model for the buckling stress is presented in Figure 2.

The problem is a straight, cylindrical penis as a stiff compression member, elastically clamped to the body and supported by a flexible spring (restraint). The restraint is dealt with in linear and nonlinear form.

The mathematical problem is the investigation of the stability of the state of balance *φ* = 0 depending on the size of the load stress **F**, which is true to the direction. The load stress is effective in the centre of the cylinder surface (no eccentrically working force).

Physically spoken, the postbuckling behaviour and the rupture of the layer after balance branching have to be described.

It is assumed that the strength movement is “conservative” and “Dirichlet's criterion of stability” is effective.

**Figure 3:**
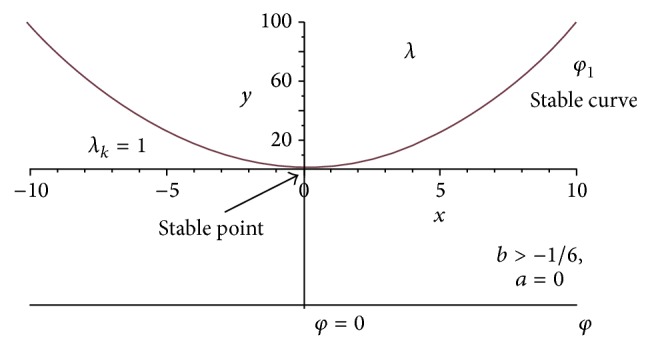
Unhazardous branching of balance in the condition of exceeding *λ*_*K*_ = 1.

**Figure 4:**
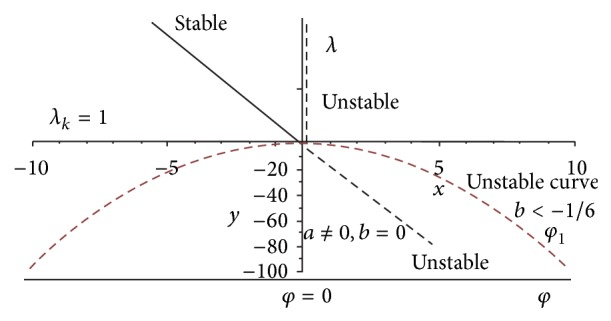
Hazardous (rupture) branching of balance at *λ*_*K*_ = 1.

The geometrical angle *φ* results from the potential energy in the neighbourhood of the state of balance as well as the potential of the external forces, whereby unhazardous balance branching (no rupture) and hazardous balance branching (rupture) arise. Figures 3 and 4 represent these results graphically.

The states of balance and the neighbouring deflected states of balance result from the following equation after algebraic simplifications and differentiations

(2) $$\begin{array}{c} \varphi_{1}=\frac{3}{6b+Fl/k}\left[a\pm \sqrt[{2}]{a^{2}+\frac{2}{3}\left(\frac{Fl}{k}-1\right){\left(6b-\frac{Fl}{k}\right)}^{2}}\right]. \end{array}$$

The point of branching belongs then to the stable states of balance *φ* = 0, when *b* ≥ 0 is effective for a linear or overlinear spring.

An underlinear spring with *b* < −1/6 results in an unstable point of branching. The penis fibre is broken.

Stability is the result for neighbouring states of balance of a linear or nonlinear spring with symmetrical characteristic curve as long as 1 + 6*b* < 0 is effective, as *a* = 0 and *λ* > 1 are then effective.

For the underlinear spring with the condition *b* < −1/6 the point of branching is unstable. The fibre of penile tissue breaks.

For the nonlinear spring with unsymmetrical characteristic curve and the condition *b* = 0, the states of balance *φ*_1_ < 0, *λ* > 1 are stable and the states of balance *φ*_1_ > 0, *λ* < 1 are unstable. The fibre of penile tissue breaks between 1,2° and 2,94°. *λ* is the load factor (*F*/*F*_*k*_).

Table 1 [5] represents the state of rupture of the outer layers at a stress of the tunica of 120 N/cm^2^ depending on an inner pressure of the penis of 200 mmHg = 26,6 kPa and the extent of the external force as well as the angle of deviation, whereby the greater angle needs much less external force to reach the critical stress of the tunica.

Figures 3 and 4 represent the load factor deformation curves differentiated in hazardous and nonhazardous balance branches with exactly centric load.

In developing this singular penile fibre in *n* layers, the resulting fibre-load-angle for the buckling is between 1,2° and 6,4°. At a geometrical angle of 6,4° the *n*-layer breaks. This means a significant rupture of the outer layer of the penis.

## 6. Mathematical Simulation Calculations: Macroscopic Approach

The question to mathematical physics and especially the physical simulation is to calculate the distribution of forces and stress in an ideal cylinder with a central application of force from the top. In the first stage a totally filled rigid penis with 100 per cent blood filling, which means greatest solidity and greatest inner pressure of the total structure, is taken into account.

For this it is necessary to measure the geometric conditions of the penis (length and circumference). Apt starting and marginal conditions have to be set that influence the calculation net of the model. Materials and sort of stress have to be physically defined in the model. This results in the optimal net of calculation points. The tunica albuginea is the external layer of the cylinder. Its maximum stress and its localisation are calculated with various accelerations of the stress movement.

The model of the fluid-structure interaction works with the hypothesis that the totally filled rigid penis impacts on an obstacle with a certain, predefined acceleration. As the body is closed, fluid cannot issue from it. That means, the movement of the fluid in the penis is stopped with the impact and a contramovement arises, as blood is an incompressible medium. The pressure body dodges from the force and a mechanical buckling in the boundary region arises, causing a distension of the external layer (of the tunica albuginea). This maximum of stress and the distension lead to a microrupture of the tissue which is firstly not really noticed, but cicatrisation leads to a loss of elasticity. A second stress leads to even larger dodging and to the rupture of the penis under a certain geometrical angle.

For the mathematical model the following starting and marginal parameters were used. The model used in Comsol* 4.3*. is a fluid-structure-interaction model. In the first step we base the simulation on the assumption of a still straight rigid penis and the impact on a plain obstacle. As shown in Table 2.

The solution of partial differential equations with the predefined net, where the connecting points are the calculation points, results in the distribution of stress in the cylindrical penis and the localisation of the point of the highest stress. The two-dimensional distribution of stress shows the calculated distribution of force and the direction of the deviation.

**Figure 5:**
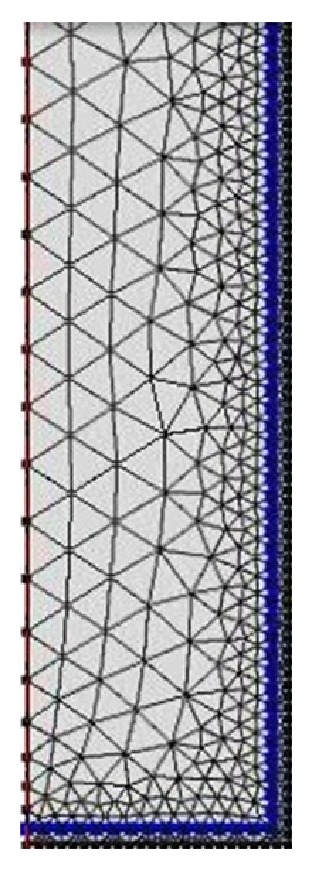
Calculation net.

**Figure 6:**
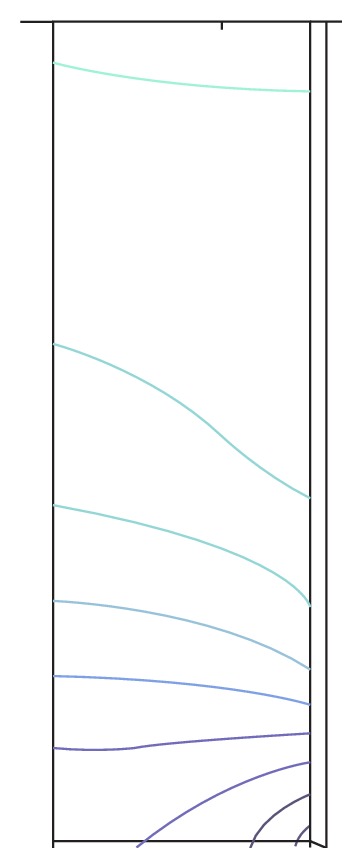
Pressure distribution in penis.

The net (Figure 5) shows the calculation points of the model. The distribution of pressure in the penis (Figure 6) shows that the pressure increases from up to down. At the point of the highest pressure, pressure and backpressure summarize to their maximum. The pressure distribution also shows more or less horizontal pressure layers, which is why penis rupture will always be horizontal rupture.

**Figure 7:**
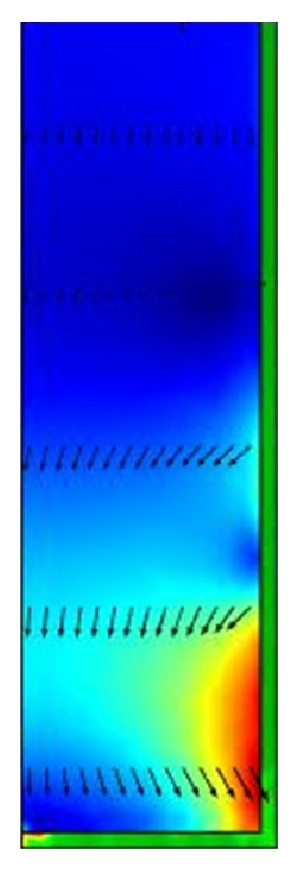
2D-stress distribution of the penis.

The highest pressure arises, where the vector arrows touch the surface of the penis (Figure 7).

**Figure 8:**
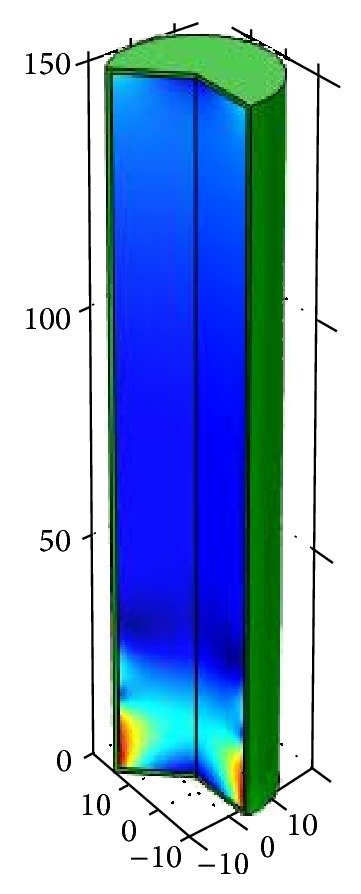
3D-stress distribution of the penis.

The three-dimensional distribution of stress (Figure 8) shows the whole cross section of the penis. Rupture of multiple fibres, if it arises, is a horizontal rupture, no rupture in longitudinal direction. The points with highest stress are the points where a notch or a rupture is to be anticipated.

The physical simulation model shows that tunica fibre rupture arises in the lower third of the nonlead penis. For simplification, the glans was not shown in this model.

## 7. Discussion

The pathogenesis of congenital penile curvature is unknown. Peyronie's disease is a poorly understood connective tissue disorder most commonly attributed to repetitive microvascular injury or trauma during intercourse [1]. Neither a special mechanism nor a mechanical explanation has been provided for this pathological process up to now.

The tunica albuginea of the corpora cavernosa is a bilayered structure with multiple sublayers. Inner layer bundles support and contain the cavernous tissue and are oriented circularly [10]. Radiating from this layer are intracavernous pillars acting as struts, which augment the septum and provide essential support to the erectile tissue. Outer layer bundles are oriented longitudinally. These fibers extend from the glans penis to the proximal crura, where they insert into the inferior pubic ramus [10].

Various models have been described before, trying to estimate penile reaction in erectile dysfunction or in Peyronie's disease [9, 11–13]. Gefen et al. developed a model that demonstrates stress concentrations around nerve roots and blood vessels due to plaques in Peyronie's disease. These stresses may irritate nerve endings or compress the vascular bed and thus cause penile deformity and/or painful erection. The model was further used to elaborate the effects of different biological or artificial materials for reconstruction of the penis following plaque removal [14].

As to the question of the differences in accuracy of the three physical models of the penis we can say the following.

There are fundamental differences in the mathematical formalisms of [13] and [12], and our model that lead to considerable differences of accuracy.

The two former models are based on hard- and software technology of the years 1998 and 2008.

The basics of each physical model are the definitions of the problem, the geometries and cubage, the differentiated assumptions of material, and the described algorithm of the problem as well as the solution of differential equations with apt marginal and starting conditions. The accuracy of the model is not only dependent on the sort of used net but also, if it is a stationary or time-dependent model. An essential point is also the calculation efficiency of the used workstation, where these problems are calculated. Good calculation efficiency guarantees also the possibility of a dense net and with this the possibility of a larger geometrical accuracy.

There are one-dimensional, two-dimensional, and three-dimensional models. In our case we used a three-dimensional model with a dense net.

The commencements as well as the aims that are described in the abstracts of the singular articles are decisive. A two-dimensional model may even offer a higher accuracy than a three-dimensional one. It depends on the questions put forward to the model.

It is of less importance, if the simulation tool is Mathematica, Matlab, Maple, Ansys, or Comsol, as the differences are caused by the used mathematical algorithm and the aims of the singular work.

The simulation tool Comsol Multiphysics is a high end simulation program of the latest generation, which means that a mechanical model can be combined in one simulation with a hydraulic model and, for example, a thermal model. This type of computational tool with the capacity of three-dimensional calculation is available only since 5 years. Previously, for example, in [13] a calculation was only displayed as a one-dimensional or two-dimensional calculation [12].

Up to now, no model was developed to describe the mechanical forces that impact on the different layers of the tunica in a physical simulation model.

Our model became a challenging topic for both faculties, in order to create a physical simulation model for the use of urological surgery to find out, for each different real case, if prophylactic measures for surgical penis correction are already necessary or not. This paper presents the first stage of the planned development of a physical simulation tool to be used by the surgeon, not the physicist.

For this reason, a human organ with its special properties had to be translated into a fitting physical model, where the physical theoretical considerations could be adequately attached to. Our starting points for developing the model were the theoretical considerations of the penis elasticity and the buckling stresses the penis and the singular fibres of penile tissue are put under. Thus the theoretical considerations lead to the calculation of the fibre-load-angles analytically.

From the analytical considerations the critical fibre-load-angles of the penis subdued to buckling stress could be determined to be 1,2° to 2,94° for the singular penile fibre and 6,4° for the *n*-layered structure of the penis. This result in itself is a novelty to the opinion in medical-surgical literature, where the fibre-load-angle of about 30° was looked on as the threshold for surgical intervention [1, 15].

The macroscopic approach represents a different base for the model and the physical consideration of it. In a fluid-structure-interaction model the penis is looked on as a whole structure, subdued to forces, pressures, and backpressures on the impact with a plain obstacle. From this model we derive the local distribution of stress in the penis and the localisation of the maximum point of pressure and stress of the outermost layers of the penis structure.

The risk of penis rupture is depending on the external force (force is mass multiplied by acceleration), the change of form, and the inner pressure.

As prospect for further publications and the development of a simple and effective tool for the medical surgical use in practice for the individual case there are different questions to be considered analytically and answered, first of all the lesser filling of the penis and, second the already asymmetrical penis or the asymmetrical, noncentric impact on an obstacle. These questions to be answered in the future with the help of physical considerations and simulation modelling will lead to different scenarios that can then be integrated into the above mentioned simulation tool.

## 8. Conclusion

Physical simulation is able to provide the surgeon with a simple instrument to calculate and forecast the risk of the individual patient, based upon dependencies of geometry of the human body.

Up to now, no model was developed to accurately describe the mechanical forces that impact on the different layers of the tunica albuginea of the penis in a modern physical simulation model.

We now demonstrate such a simulation, which is able to forecast the critical fibre-load-angles of the penis subdued to buckling stress and could be determined to be only 6,4° for the *n*-layered structure of the penis. The torsion of the fully filled and erect penis of the above mentioned angle is less than what is normally assumed to be necessary to be the starting point of Peyronie's disease.

Further modification of this model might bring a deeper insight into the mechanical and physical mechanisms of induction of Peyronie's disease.

## Figures and Tables

**Table 1:** 

Geometric angle *φ*	External force *F*
1°	2,90 kN
2°	2,65 kN
3°	2,40 kN
4°	2,15 kN
5°	1,90 kN
6°	1,65 kN

**Table 2:** 

The length of the penis in erect state (cylinder)	140 mm
Diameter of penis in erect state	30 mm
Circumference of penis in erect state	94,2 mm
Mass of penis (estimated)	0,2 kg
Tunica albuginea width in erect state	between 1 mm and 2 mm
Maximum inner pressure—100 per cent filling	200 mmHg = 26,6 kPa
Density of blood	1,055 g/cm³
Highest possible stress of the tunica	120 N/cm^2^
Velocity of movement	10 cm/s
